# Sex-Specific Social Effects on Depression-Related Behavioral Phenotypes in Mice

**DOI:** 10.3390/life11121327

**Published:** 2021-12-01

**Authors:** Seona D. Patel, Lindsay P. Cameron, David E. Olson

**Affiliations:** 1 Department of Chemistry, University of California, Davis, One Shields Avenue, Davis, CA 95616, USA; seopatel@ucdavis.edu; 2 Neuroscience Graduate Program, University of California, Davis, Davis, CA 95618, USA; lcameron@ucdavis.edu; 3 Department of Biochemistry & Molecular Medicine, School of Medicine, University of California, Davis, 2700 Stockton Blvd, Suite 2102, Sacramento, CA 95817, USA; 4 Center for Neuroscience, University of California, Davis, 1544 Newton Ct, Davis, CA 95618, USA

**Keywords:** depression, stress, social effects, chronic corticosterone, forced swim test, sex differences

## Abstract

Social interaction and empathy play critical roles in determining the emotional well-being of humans. Stress-related depression and anxiety can be exacerbated or mitigated depending on specific social conditions. Although rodents are well known to exhibit emotional contagion and consolation behavior, the effects of group housing on stress-induced phenotypes in both males and females are not well established. Here, we investigated how the presence of stressed or unstressed conspecifics within a cage impact depression-related phenotypes. We housed male and female C57BL/6J mice in same-sex groups and subjected them to either gentle handling (GH) or the daily administration of corticosterone (CORT) for 10 days. The GH and CORT treatment groups were divided into cages of unmixed (GH or CORT) and mixed (GH and CORT) treatments. Depression-related phenotypes were measured using the forced swim test (FST) and sucrose preference test (SPT). We found that mixed housing alters FST behavior in a sex-specific manner. Male mice given chronic corticosterone (CORT) that were housed in the same cage as gently handled animals (GH) exhibited increased immobility, whereas GH females housed with CORT females demonstrated the opposite effect. This study underscores the importance of social housing conditions when evaluating stress-induced behavioral phenotypes and suggests that mixed cages of GH and CORT animals yield the greatest difference between treatment groups. The latter finding has important implications for identifying therapeutics capable of rescuing stress-induced behavioral deficits in the FST.

## 1. Introduction

Major depressive disorder (MDD) is a leading cause of disability, affecting more than 264 million people worldwide [1]. A combination of genetic susceptibility, psychosocial factors, alterations in neurotransmission, changes in hormone levels, and structural plasticity influence the development of depression. Despite extensive clinical and preclinical research, the pathophysiology of depression is not completely understood, though many studies have demonstrated that exposure to chronic stress may contribute to the precipitation or exacerbation of depression [2,3,4].

A variety of animal models have been developed to understand the influence of chronic stress on depressive symptomatology and include chronic unpredictable mild stress [5], chronic restraint stress [6], and chronic social defeat stress [7]. As these models are extremely labor intensive and can produce highly variable outcomes, simpler protocols with improved replicability across experimenters have been sought. The chronic administration of corticosterone has emerged as a particularly attractive method due to its simplicity and ability to induce many of the behavioral and neurobiological changes in rodents that are relevant to identifying antidepressant treatments [8,9]. Corticosterone is a glucocorticoid that is released from the adrenal glands of rodents in response to stress and performs an analogous function to cortisol in humans. Rodents subjected to chronic corticosterone administration display increased immobility in the FST [10,11,12], increased anhedonia as measured by sucrose intake [10,11,13,14], decreased weight gain [12,13,14], anxiogenic behavior in the open field [13,14], cognitive impairment in spatial learning [14], and dysregulated hypothalamic–pituitary–adrenal (HPA) axis function [12,14]. Protocols for administering corticosterone vary by dosage, route of administration, duration of exposure to corticosterone, and vehicle used to solubilize corticosterone. These variables can lead to different effects observed in behavioral tasks relevant to neuropsychiatric disorders [15]. The duration of corticosterone administration typically varies from 1–9 weeks, and the most common routes of administration are via drinking water, implantable pellets, subcutaneous injections, or intraperitoneal (ip) injections [15].

Although chronic corticosterone administration can successfully induce several depressive-like phenotypes, one variable that can impact behavioral outcomes is how the animals are housed. For example, individual and group housing are known to differentially impact responses to stress [16,17]. Singly housed animals subjected to chronic corticosterone or vehicle treatment for three weeks exhibited an increase in anxiety-like behavior compared to group housed animals, independent of treatment [15]. Furthermore, compared to group-housed controls, singly housed animals exhibited significantly increased immobility in the FST [18], decreased social interaction [18], increased anxiety-like behavior [19], higher levels of corticosterone [19], reduced levels of BDNF in the brain [19], and altered immunoendocrine responses to mild acute stress [20]. It is clear that single housing is a powerful insult that is sufficient to induce depressive phenotypes in social animals such as mice.

To study the effects of stress in the context of an animal’s social environment, group housing is often employed. It is now increasingly appreciated that rodents display empathy-like behaviors such as observational fear [21], emotional contagion for pain [22,23], and pro-social consolation [24]. In addition, it has been shown that rodents engage in behaviors to help distressed conspecifics based on empathetic concern [24]. Mice that are housed in the same room as mice subjected to pain will exhibit hyperalgesia [22]. Although it is unknown whether mice that observe stressed cagemates exhibit increased depressive phenotypes, it is reasonable to hypothesize that housing stressed and unstressed animals within the same cage may impact phenotypes relevant to depression.

In studies that employ group housing, it is not known whether mixed treatment housing impacts depression-like phenotypes. However, a previous study demonstrated that interactions between morphine-treated and drug-naïve cagemates decreased and increased the abuse potential of opioids in these groups, respectively [25]. Thus, mixed treatment housing can have a profound impact on the addiction-related effects of morphine. Moreover, the stress status of cagemates has been shown to influence social buffering effects in rats [26], which emphasizes the need to consider mixed treatment housing in experimental designs for depression research. Here, we use the FST to demonstrate that housing conditions play a pivotal role in determining the behavioral phenotypes of male and female animals exposed to chronic corticosterone (CORT) or gentle handling (GH) only.

## 2. Materials and Methods

### 2.1. Animals

Male and female C57BL/6J mice (9–10 weeks old) were obtained from the Jackson Laboratory (Sacramento, CA, USA) and housed in a vivarium at the University of California, Davis. Mice were allowed to habituate to the vivarium for at least one week upon arrival before the initiation of the experiments. Mice of the same sex were housed in groups of five per cage in a temperature- and humidity-controlled room maintained on a 12 h light/dark cycle (lights were turned on at 7:00 a.m. and turned off at 7:00 p.m.) with ad libitum access to food and water. Experiments were conducted between the hours of 8:00 a.m. and 4:00 p.m.

### 2.2. Chronic Corticosterone Administration

Corticosterone (Spectrum Chemical, Gardena, CA, USA) was dissolved in DMSO (Spectrum Chemical, Gardena, CA, USA) to obtain a 10 mg/mL solution. After 1 week of habituation to the vivarium, each mouse was weighed and either gently handled (GH) for 1–2 min or given a 20 mg/kg intraperitoneal injection of corticosterone (CORT) dissolved in 100% DMSO (2 mL/kg) on days 1–10. Solutions of CORT were prepared fresh daily. Extensive studies were conducted to solubilize CORT with a variety of vehicles including saline solutions containing Kolliphor, Tween, or DMSO. None proved satisfactory even after sonication. Therefore, we opted to administer CORT using a low-volume, 100% DMSO vehicle. This amount of DMSO is well below the LD_50_ of DMSO in mice when administered via ip injection and was approved by the UC Davis IACUC. Over the course of our studies, no animals perished.

### 2.3. Sucrose Preference Test (SPT)

On day 11, anhedonia was assessed via the SPT. Mice were habituated to the experimentation room in the absence of water for 2 h prior to the start of the experiment in their home cages. After the habituation period, mice were individually housed and given one bottle of water and one bottle of 1% sucrose in water. Solutions of sucrose were made fresh. Sucrose and water intake were monitored for 4 h between 9:00 a.m. and 1:00 p.m. Sucrose preference was calculated as the amount of sucrose consumed minus the amount of water consumed, divided by the total amount of liquid consumed. A score approaching “0” indicates no preference for sucrose, a positive score indicates a preference for sucrose, and a negative score indicates a preference for water. Data points were omitted if bottles leaked during the test.

### 2.4. Forced Swim Test (FST)

On day 12, the FST was conducted using a clear plexiglass cylinder that was 40 cm tall and 20 cm in diameter, filled with 23 ± 1 °C water to a height of 30 cm. Mice spent 6 min in the water, and the time spent immobile was scored for the last 4 min of the session. The experiments were divided into two cohorts of animals and conducted on different days. Experiments were video-recorded and manually scored by a trained experimenter blinded to treatment conditions. Immobility was defined as passive floating or remaining motionless with no activity other than that needed to keep the animal’s head above water. Fresh water was used for each test.

### 2.5. Statistical Analyses

Data analyses for the FST and SPT were performed by experimenters blinded to treatment conditions. Statistical analyses were performed using GraphPad Prism (version 9.1.2). Data were excluded from the SPT analysis whenever bottles were clearly leaking. No data were excluded from the FST tests.

## 3. Results

### 3.1. Chronic CORT Treatment Is Associated with Lower Body Weight

**Figure 1:**
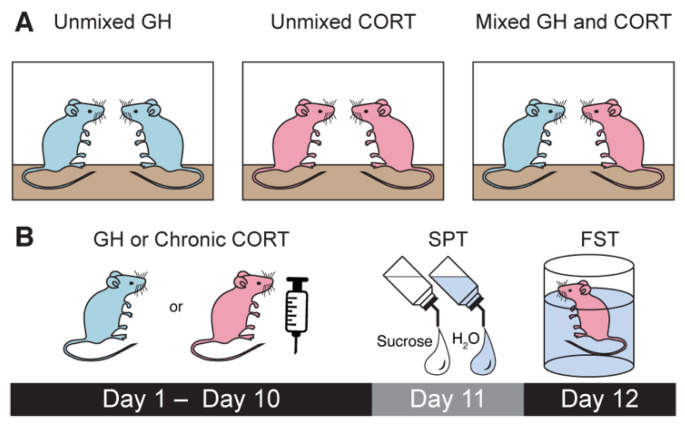
Experimental design for studying the effects of mixed housing on stress-related behavioral phenotypes. (**A**) Schematic depicting the three types of housing conditions used in these studies. (**B**) Schematic depicting the timeline for the chronic corticosterone administration and behavioral experiments relevant to depression.

To investigate the effects of mixed treatment group housing on chronically stressed and unstressed male and female mice, we employed chronic corticosterone administration as a model of chronic stress. Animals were exposed to either gentle handling (GH) or daily intraperitoneal injections (20 mg/kg) of corticosterone in DMSO (CORT) for a total of 10 days (Figure 1A,B). Half of the male and female GH mice were housed with GH mice only (unmixed GH), whereas the other half were housed with CORT mice (mixed GH). Mixed housing consisted of either three GH and two CORT or two GH and three CORT animals per cage. All animals were housed in cages with animals of the same sex. Similarly, half of the male and female CORT mice were housed with CORT mice only (unmixed CORT), and the other half were housed with GH mice (mixed CORT) (Figure 1A,B).

**Figure 2:**
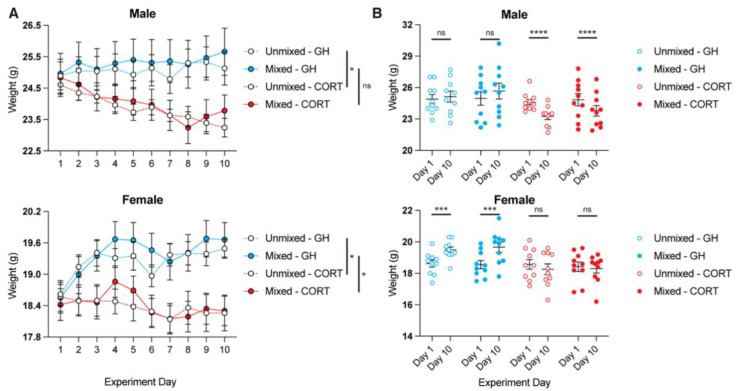
Mice treated with chronic corticosterone have lower body weights. (**A**) Animals were weighed each day during chronic corticosterone administration. Statistical differences were observed between GH and CORT groups on Day 10 (two-way ANOVA with a Tukey post-hoc test, males *F*_3,27_ = 5.367, females *F*_3,27_ = 6.768; males, unmixed GH vs. unmixed CORT, *p* = 0.050; males, mixed GH vs. mixed CORT, *p* = 0.052; females, unmixed GH vs. unmixed CORT, *p* = 0.027; females, mixed GH vs. mixed CORT, *p* = 0.013). (**B**) In males, CORT decreased weight from Day 1 to Day 10 (multiple paired *t*-tests; unmixed CORT, *p* = 0.000085; mixed CORT, *p* = 0.00083). In females, CORT prevented weight gain from Day 1 to Day 10 (multiple paired *t*-tests; unmixed GH, *p* = 0.00019; mixed GH, *p* = 0.00030). * *p* < 0.05, ** *p* < 0.01, *** *p* < 0.001, **** *p* < 0.0001, ns = not significant.

Following the administration of corticosterone for 10 days, mixed and unmixed CORT males exhibited significantly decreased body weights on Day 10 compared to their baseline body weights measured on Day 1 (Figure 2A,B). Similar changes were observed for mixed and unmixed groups. There were no statistical differences between Day 1 and Day 10 body weights for GH males. Unlike GH males, GH females gained a significant amount of weight over the course of 10 days (Figure 2A,B). This increase in body weight was completely prevented by the chronic administration of corticosterone. As with the males, housing conditions did not seem to impact the effects of corticosterone on body weight.

The chronic administration of corticosterone produces sex-specific changes in body weight, with corticosterone inducing weight loss and preventing weight gain in males and females, respectively. Regardless, the chronic administration of corticosterone results in statistical differences in weight between GH and CORT treatment groups on Day 10 (Figure 2A), indicating a robust response to chronic stress. Moreover, housing conditions (i.e., mixed vs. unmixed) do not seem to impact the effects of corticosterone on weight.

### 3.2. Mixed Treatment Group Housing Has No Impact on Sucrose Preference

**Figure 3:**
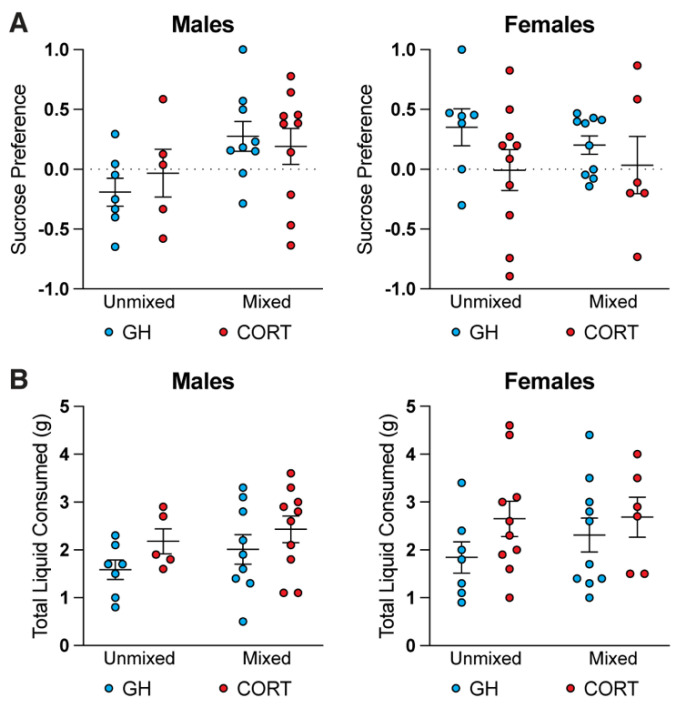
Mixed treatment group housing does not impact sucrose preference. (**A**) Treatment group does not significantly impact the sucrose preference of either males or females (two-way ANOVA with a Tukey post-hoc test, males housing *F*_1,27_ = 5.121, males treatment *F*_1,27_ = 0.06003, females housing *F*_1,29_ = 0.1097, females treatment *F*_1,29_ = 2.677). (**B**) Treatment group does not significantly impact the total liquid consumed for either males or females (two-way ANOVA with a Tukey post-hoc test, males housing *F*_1,27_ = 1.320, males treatment *F*_1,27_ = 2.970, females housing *F*_1,29_ = 0.4291, females treatment *F*_1,29_ = 2.388). No statistically significant results were observed.

To explore the effects of chronic stress and mixed treatment housing on behavioral phenotypes relevant to depression, animals were subjected to the SPT and FST on Days 11 and 12, respectively (Figure 1B). In the SPT, there was no statistical difference between housing conditions for either males or females (Figure 3A). Unmixed female, mixed female, and mixed male CORT groups exhibited slight reductions in sucrose preference compared to GH controls, though these differences were not statistically significant. Neither GH nor CORT unmixed males displayed a sucrose preference, perhaps due to the short duration of the test (4 h). Rodents tend to drink more liquids at night during the active phase [27], and thus, more robust sucrose preferences can be obtained when animals are given access to sucrose for longer periods of time, particularly at night [28]. Finally, the chronic administration of corticosterone tended to increase total liquid consumption in all groups (Figure 3B), though these trends were not statistically significant compared to the GH controls. Corticosterone administration has been shown to increase water consumption in rodents within the first two weeks of administration [29,30], suggesting that the CORT administration was appropriate.

### 3.3. Mixed Treatment Group Housing Produces Sex-Specific Effects in the Forced Swim Test

**Figure 4:**
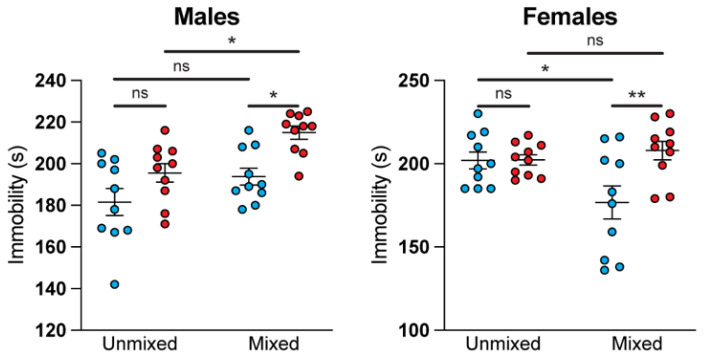
Mixed treatment group housing produces sex-specific effects in the FST. Chronic corticosterone increases immobility to a greater extent when male mice are subjected to mixed treatment group housing (two-way ANOVA with a Tukey post-hoc test: males housing *F*_1,36_ = 11.27, males treatment *F*_1,36_ = 13.99, females housing *F*_1,36_ = 2.345, females treatment *F*_1,36_ = 5.995). In contrast, chronic corticosterone does not increase immobility in female mice. However, mixed housing produces an antidepressant-like effect in female GH mice (2-way ANOVA). * *p* < 0.05, ** *p* < 0.01, ns = not significant.

On Day 12, mice were subjected to the FST. Mixed male CORT mice exhibited increased immobility compared to mixed GH mice (Figure 4). Although a similar trend between GH and CORT mice was observed for unmixed animals, the difference was not statistically significant (*p* = 0.17). Moreover, mixed housing seems to potentiate an immobility phenotype, as mixed CORT males exhibited significantly greater immobility than unmixed CORT males. Taken together, this suggests that mixed housing results in larger differences between GH and CORT treatment groups in males with respect to FST behavior.

Although the chronic administration of corticosterone increases immobility in male mice, it does not produce this same effect in female mice (Figure 4). There is no statistical difference between unmixed GH and unmixed CORT female mice. Furthermore, mixed CORT female mice were not statistically different from either unmixed treatment group. Interestingly, we observed that female mixed GH mice spent significantly less time immobile than unmixed GH mice. As a result, female mixed GH and mixed CORT mice were statistically different from each other.

### 3.4. Ratio of CORT/GH Animals in Mixed Housing Does Not Impact FST or SPT Results

**Figure 5:**
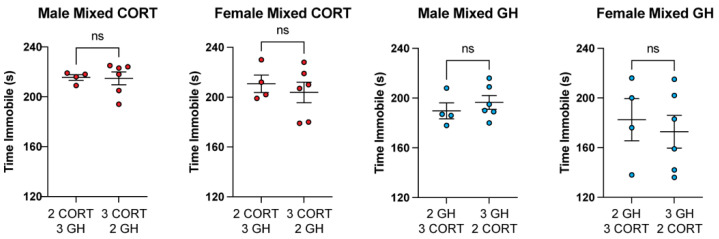
Different ratios of stressed to unstressed animals in a cage do not impact FST behavior. Housing two or three CORT animals with three or two GH animals, respectively, has no effect on immobility in males or females in the FST (unpaired *t*-test). ns = not significant.

**Figure 6:**
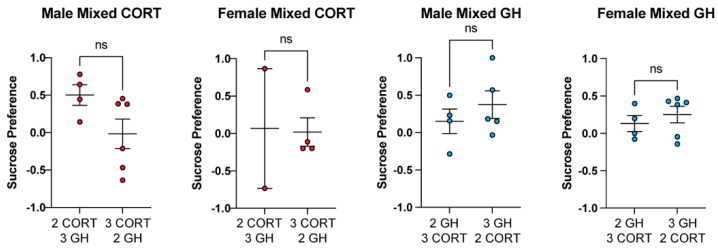
Different ratios of stressed to unstressed animals in a cage do not impact SPT behavior. Housing two or three CORT animals with three or two GH animals, respectively, has no effect on sucrose preference in males or females (unpaired *t*-test). ns = not significant.

In mixed housing, cages either housed two GH and three CORT animals, or two CORT and three GH animals. In the FST, we found no statistical differences between mixed animals housed with three CORT animals and mixed animals housed with two CORT animals (Figure 5). Similar results were observed for the SPT (Figure 6). Further studies are necessary to delineate whether having a more drastic ratio of stressed to unstressed animals within a cage (e.g., 1:4 or 4:1) alters depressive phenotypes in mixed animals.

## 4. Discussion

This study demonstrates that exposure to different social housing conditions can modulate immobility in the FST in a sex-specific manner. Typically, researchers use some form of chronic stress to induce a deficit in FST behavior in the hopes of rescuing the deficit with novel therapeutics. Here, we employed the chronic administration of corticosterone as our stressor given that it is widely used due to its operational simplicity and reproducibility. Similar to other studies [12,13,14], our results demonstrate that CORT mice exhibit lower body weights compared to GH controls.

The chronic corticosterone paradigm, similar to many other chronic stress models, demonstrates a degree of phenotypic variability depending on the experimental design employed. The effects of chronic corticosterone on measures related to anxiety and depression can be impacted by the method of administration [31], dosage of corticosterone [12], drug formulation used to solubilize CORT [32], as well as mouse strain [33] and sex [28]. An often-overlooked variable is housing conditions. In this study, we found that housing conditions did not affect body weights or sucrose preference following the chronic administration of corticosterone, but they did have profound effects on FST behavior. We chose to employ a 20 mg/kg ip injection of corticosterone over 10 days as a moderate stressor because we were concerned that a more intense chronic corticosterone protocol (e.g., 40 mg/kg or 20 mg/kg for 21 days [12]) might mask the effects of mixed housing on depressive phenotypes.

Although the chronic corticosterone paradigm has been shown to generally produce depression-related phenotypes such as increased immobility in the FST and decreased sucrose preference, different protocols have demonstrated varying behavioral results and it is not uncommon for chronic corticosterone to impact one behavioral measure while leaving another unchanged [15]. For example, a recent study demonstrated that in the SPT, there was no difference between control animals and those treated with corticosterone chronically [32]. However, the groups did exhibit statistical differences in the FST [32]. Although animals in this study were singly housed, these results are consistent with what we report here.

One possible explanation for why we observed an effect of mixed housing on FST but not SPT behavior could be related to the different circuits involved in these behaviors. Whereas sucrose preference appears to involve neurons in the nucleus accumbens that project to the ventral pallidum, [34], swimming behavior in the FST has been shown to be mediated by a projection from the medial prefrontal cortex to the dorsal raphe nucleus [35]. Another possibility is that some animals are resilient to the effects of chronic corticosterone on sucrose consumption. To test this hypothesis, future studies should perform a baseline SPT prior to corticosterone administration. Animals that do not exhibit a decrease in their sucrose preference following chronic corticosterone could be categorized as resilient and analyzed separately. However, a similar strategy cannot be used for the FST, as this test is considered a stressful paradigm [36], and thus, likely to impact GH animals.

A common method for administering corticosterone is via drinking water. These conditions necessitate unmixed housing for GH and CORT mice. However, our results in the FST indicate that unmixed housing results in only modest differences between male GH and CORT mice (*p* = 0.17) and no differences between female GH and CORT mice (*p* = 0.99). In sharp contrast, mixed housing produced robust differences between GH and CORT treatment groups in both males (*p* = 0.015) and females (*p* = 0.0080).

Often, researchers neglect to report how treatments are assigned within and between cages. The experiments described here suggest that this variable can play a critical role in determining behavioral outcomes, further supporting the impact of social interaction on behavioral phenotypes relevant to neuropsychiatric disorders. For researchers interested in using the FST to develop novel antidepressants, our results also suggest that mixed housing will create larger differences between GH and CORT groups, making it easier to identify compounds with desirable antidepressant-like properties and potentially improving replicability.

## Data Availability

Not applicable.
